# Social Plasticity Relies on Different Neuroplasticity Mechanisms across the Brain Social Decision-Making Network in Zebrafish

**DOI:** 10.3389/fnbeh.2016.00016

**Published:** 2016-02-16

**Authors:** Magda C. Teles, Sara D. Cardoso, Rui F. Oliveira

**Affiliations:** ^1^Instituto Superior de Psicologia Aplicada-Instituto UniversitárioLisboa, Portugal; ^2^Instituto Gulbenkian de CiênciaOeiras, Portugal; ^3^Champalimaud Neuroscience Programme, Champalimaud Centre for the UnknownLisboa, Portugal

**Keywords:** behavioral flexibility, social competence, social behavior, neuroplasticity, synaptic plasticity, neurogenesis

## Abstract

Social living animals need to adjust the expression of their behavior to their status within the group and to changes in social context and this ability (social plasticity) has an impact on their Darwinian fitness. At the proximate level social plasticity must rely on neuroplasticity in the brain social decision-making network (SDMN) that underlies the expression of social behavior, such that the same neural circuit may underlie the expression of different behaviors depending on social context. Here we tested this hypothesis in zebrafish by characterizing the gene expression response in the SDMN to changes in social status of a set of genes involved in different types of neural plasticity: *bdnf*, involved in changes in synaptic strength; *npas4*, involved in contextual learning and dependent establishment of GABAergic synapses; neuroligins (*nlgn1* and *nlgn2*) as synaptogenesis markers; and genes involved in adult neurogenesis (*wnt3* and *neurod*). Four social phenotypes were experimentally induced: Winners and Losers of a real-opponent interaction; Mirror-fighters, that fight their own image in a mirror and thus do not experience a change in social status despite the expression of aggressive behavior; and non-interacting fish, which were used as a reference group. Our results show that each social phenotype (i.e., Winners, Losers, and Mirror-fighters) present specific patterns of gene expression across the SDMN, and that different neuroplasticity genes are differentially expressed in different nodes of the network (e.g., BDNF in the dorsolateral telencephalon, which is a putative teleost homolog of the mammalian hippocampus). Winners expressed unique patterns of gene co-expression across the SDMN, whereas in Losers and Mirror-fighters the co-expression patterns were similar in the dorsal regions of the telencephalon and in the supracommissural nucleus of the ventral telencephalic area, but differents in the remaining regions of the ventral telencephalon. These results indicate that social plasticity relies on multiple neuroplasticity mechanisms across the SDMN, and that there is not a single neuromolecular module underlying this type of behavioral flexibility.

## Introduction

Social plasticity (aka “social competence,” Taborsky and Oliveira, 2012), defined as the ability to adaptively change the expression of social behavior according to previous experience and to social context, is ubiquitous among group-living animals. The effect of previous social experience on subsequent behavior has been described in a wide range of animals both in competitive and cooperative contexts, as illustrated by experience-dependent winner-loser effects (Hsu et al., 2006; Rutte et al., 2006) and reciprocity of cooperative behavior (Bshary and Grutter, 2006; Rutte and Taborsky, 2007), respectively. Similarly, the effect of social context on social behavior can be illustrated by different social phenomena present in many different species, such as “dear enemy”/“nasty neighbors” effects (Temeles, 1994; Müller and Manser, 2007), audience effects (Doutrelant et al., 2001; Pinto et al., 2011), social eavesdropping (Oliveira et al., 1998; Earley, 2010), and mate choice copying (Witte and Ryan, 2002). All these examples illustrate how social plasticity allows animals to optimize their social relationships in relation to the complexities of their social environment, and therefore it should be seen as a key determinant of their Darwinian fitness (Oliveira, 2009; Taborsky and Oliveira, 2012). Given its biological relevance there are important implications of social plasticity both for the study of behavior and evolution. First, given the prominent role of the nervous system in orchestrating flexible responses to cues that signal environmental change, the understanding of the mechanisms underlying social plasticity is crucial for understanding behavior and brain evolution (e.g., Dunbar and Shultz, 2007). Secondly, social plasticity can be seen either as a constraint or as a motor of evolution depending on environmental heterogeneity, availability of cues that signal environmental changes and the costs and limits of plasticity (DeWitt et al., 1998; Price et al., 2003; Pigliucci, 2005). Knowledge of the genetic architecture and the proximate mechanisms underlying social plasticity is crucial to understanding its costs, limits and evolutionary consequences. Therefore, the study of the neuromolecular mechanisms of social plasticity should be seen as a central topic in current behavioral research.

In terms of proximate mechanisms, social plasticity can be conceptualized as reversible shifts between behavioral states (i.e., the consistent expression of a set of behaviors) in response to relevant social information, which are paralleled by shifts between neurogenomic states (i.e., the expression of co-regulated gene sets, Cardoso et al., 2015). Thus, at the molecular level socially-driven behavioral flexibility must rely on neuronal activity-dependent mechanisms that change the neurogenomic state of the brain in response to perceived social stimuli (Cardoso et al., 2015). For example, the activation (e.g., phosphorylation) of relevant proteins (e.g., cAMP response element-binding, CREB), which then act as transcription factors (e.g., pCREB), may lead to the expression of immediate early genes (IEG). These IEGs can encode other transcription factors (e.g., *c-fos, egr-1*) or synaptic proteins (e.g., Arc, Homer1a), hence acting as neuromolecular switches that change the neurogenomic state of the brain (Aubin-Horth and Renn, 2009; Wolf and Linden, 2012; Cardoso et al., 2015).

The neuromolecular mechanisms potentially involved in social plasticity discussed above must be in action at brain regions relevant for the expression of social behavior. Recently it has been proposed the occurrence of an evolutionary conserved social decision making network (SDMN) in vertebrate brains, that regulates a variety of social behaviors, from aggression, to mating, and parental care (O'Connell and Hofmann, 2011, 2012). According to this proposal, the SDMN is composed by two interconnected neural circuits, the social behavior network (Newman, 1999; Goodson, 2005) and the mesolimbic reward system (O'Connell and Hofmann, 2011). Together these two circuits include a core collection of nuclei that are reciprocally connected and that encode information in a distributed fashion, such that the expression of a specific social behavior is better explained by the overall pattern of activation of the network rather than by the activity of a single node (Goodson and Kabelik, 2009). However, whereas the homologies (at least partial) across vertebrates for the nodes of the social behavior network and their involvement in the expression of social behavior have been firmly established across vertebrates (Newman, 1999; Goodson, 2005; Goodson and Kingsbury, 2013), the role of the mesolimbic reward system in social behavior and the occurrence of homologous network nodes have not yet been established beyond mammals (Goodson and Kingsbury, 2013). For example, an homologous area of the mammalian ventral tegmental area of the midbrain (VTA), which is known to play a key role in neural processes underlying reward in mammals (e.g., Lammel et al., 2012), remains to be identified in teleost fish (Tay et al., 2011). On the other hand, the involvement of other components of the mesolimbic reward system, namely the medial zone of the dorsal telencephalic area (Dm) and the lateral zone of the dorsal telencephalic area (Dl), which are putative homologs of the mammalian basolateral amygdala and of the hippocampus, respectively (Broglio et al., 2005), in non-mammalian social behavior has recently been shown (e.g., fish: Maruska et al., 2013a,b; Teles et al., 2015). Thus, even with the abovementioned caveats, the SDMN is still a promising framework for testing hypotheses related to the neural mechanisms underlying social behavior in vertebrates.

If one considers the SDMN, or a similar network structure, as the underlying neural mechanism for the expression of social behavior, not only temporal but also spatial changes in gene expression across its nodes may contribute for the differential activation of the network, and concomitantly to the generation of different behavioral states. Given that, at the molecular level different neural plasticity mechanisms may be in action, it is important for the understanding of the genetic architecture of social plasticity to assess if they occur independently at each of the nodes of the SDMN. Previous studies have already established that behavioral transitions are associated with changes in the pattern of IEGs expression across the SDMN. In the African cichlid fish *Astatotilapia burtoni*, the opportunity to rise in social rank increased the expression of IEGs in all studied SDMN nuclei, whereas descend in social rank showed a distinct activation across the SDMN for the IEGs *c-fos* and *egr-1* (Maruska et al., 2013a,b). In zebrafish winners and losers of a single social interaction also exhibit acute changes in the pattern of expression of *c-fos* and *egr-1* across the SDMN suggestive of socially-driven changes in functional connectivity among the nodes of these network (Teles et al., 2015). However, these studies have only focused on the expression of IEGs, and the hypothesis that different neuromolecular mechanisms involved in neuroplasticity may act independently at each of the nodes of the SDMN remains to be tested.

In this paper we used zebrafish (*Danio rerio*) to study socially-driven changes in behavioral state as a model to study social plasticity. Specifically, we assessed how induced changes in male zebrafish social status impact the expression of a set of genes known to be involved in different types of neuroplasticity across different nodes of the SDMN. Male zebrafish express experience-dependent dominance behavior, such that dominant and subordinate individuals express different behavioral profiles (Paull et al., 2010), and the outcome of a single agonistic interaction in socially isolated individuals is enough to induce experience-dependent shifts in status-dependent behavioral state (Oliveira et al., 2011). We used an established agonistic paradigm under which male zebrafish socially isolated overnight consistently express aggressive behavior and a dominance relationship is established with a clear winner and a clear loser (Teles et al., 2013). We consider that winners and losers experience a change in social status in opposite directions (gain and loss, respectively), given their different perceived ratio of the aggressive acts given and received during the interaction. Two control treatments were also included in the experiment: (1) non-interacting fish that were kept in social isolation for the same amount of time; and (2) fish that fought their own image on a mirror, and therefore despite expressing aggressive behavior did not experience a change in social status, since the number of aggressive acts performed equals those perceived in the opponent (mirror-image). The non-interacting control treatment provides a reference group, whereas a comparison of real-opponent fighters (i.e., Winners and Losers) with the Mirror-fighters will allow us to distinguish gene responses associated with a behavioral shift (present in winners and losers) from those related to the expression of fighting behavior (also present in mirror fighters, but where no status shift occurred). In summary our interpretation of possible results is the following:

1. changes in gene expression between Winners/Losers and non-interacting fish that are not present in Mirror-fighters are associated with changes in social status (i.e., social plasticity);
2. changes in gene expression between Winners/Losers and non-interacting fish also present in Mirror-fighters reflect aspects of fighting behavior and are not associated with changes in social status;
3. changes in gene expression between Mirror-fighters and non-interacting fish that are not present in Winners/Losers reflect their fighting behavioral state and are not associated with a shift in social status.

The following genes were used as markers of different types of neuroplasticity: brain-derived neurotrophic factor (*bdnf*), involved in changes in synaptic plasticity by increasing synaptic strength in response to excitatory transmission (Leal et al., 2014); neuronal PAS domain protein 4a (*npas4*), involved in homeostatic plasticity, by enhancing inhibitory synapses in response to excitatory transmission (Lin et al., 2008); neuroligin 1 (*nlgn1*) and neuroligin 2 a/b (*nlgn2*), as synaptogenesis markers (Krueger et al., 2012); and neuronal differentiation 1 (*neurod*) and wingless-type MMTV integration site family, member 3 (*wnt3*) as indicators of neurogenesis (Aimone et al., 2014). Importantly all these genes have already been identified in zebrafish (Hashimoto and Heinrich, 1997; Rissone et al., 2010; Klarić et al., 2014) and some of them have been shown to be involved in similar functions (e.g., *wnt3* and neurod regulation of neurogenesis, Clements et al., 2009; Ochocinska and Hitchcock, 2009). Plasma cortisol levels were also measured to detect rapid physiological changes.

## Materials and methods

### Animals

Our study subjects consisted of 45 adult wild-type (AB) zebrafish males bred and held at Instituto Gulbenkian de Ciência (IGC, Oeiras, Portugal). Fish were kept in a recirculating system (ZebraTec, 93 Tecniplast), at 28°C with a photoperiod of 14L:10D in mixed tanks. Water system was monitored for nitrites (< 0.2 ppm), nitrates (< 50 ppm) and ammonia (0.01–0.1 ppm). Conductivity and pH were maintained at 700 μSm and 7 respectively. Fish were fed twice a day with *Artemia salina* in the morning and commercial food flakes in the afternoon, except on the day of the experiments.

### Experimental procedure

A behavioral paradigm previously used to study agonistic interactions (Oliveira et al., 2011; Teles et al., 2013) was followed. In brief, males were paired in size-matched dyads [standard length (mean ± SEM) = 3.78 ± 0.03 cm; body mass (mean ± SEM): 0.4 ± 0.00 g], and placed in a experimental arena (5 × 8 × 6 cm), which was divided in two compartments by one or more removable opaque partition(s) (see below). Members of each dyad were kept overnight in visual isolation, each one on each compartment of the experimental arena. After this period, one or more of the partitions were removed and the fish were allowed to interact for 30 min. Three social treatments were used: (1) fighting a real-opponent conspecific, where there was a single opaque PVC partition separating the two fish, which was removed; (2) fighting their own image on a mirror, where there were two mirrors, each facing one of the compartments, behind the opaque partitions; the partitions were removed to uncover the mirrors but a central partition separating the two compartments remained in place; and (3) no agonistic interaction, where there were three central opaque partitions, and only the outer two were removed (to control for putative stress effects of handling partitions in the experimental tanks). These social treatments generated four social behavior states: winners (W, *n* = 12) and losers (L, *n* = 11) of the real opponent interaction; mirror-fighters (M, *n* = 12); and non-interacting fish (i.e., visual isolation, I, *n* = 10). All animals were tested in pairs in order to give them access to conspecific odors, which would otherwise only be present in real opponent dyads, therefore avoiding confounding effects of putative chemical cues in the comparisons between treatments. Behavioral interactions were video-recorded for subsequent behavioral analysis. Two hours after the end of the interaction, animals were killed with an overdose of tricaine solution (MS222, Pharmaq; 500–1000 mg/L), and blood collect for hormonal analysis. The choice of 2 h post-interaction for the biological sampling is justified by the fact that this study was focused on the expression of late expression genes putatively involved in neural plasticity (i.e., *neurod, wnt3, nlgn1, nlgn2*) and the two immediate early genes (i.e., *bdnf, npas4*) also included in the candidate genes list also have a time course of expression compatible with this sampling point (*bdnf*: Zafra et al., 1990; Saha et al., 2011; Taylor et al., 2011; *npas4*: Ramamoorthi et al., 2011).

### Blood collection and hormone analysis

Blood samples were collected from the caudal vein using a 300 μl syringe with a 30G needle. Blood was subsequently centrifuged at 1000 × g for 10 min, and the plasma collected into a new tube, diluted in EIA buffer (1:50) and stored at −20°C until further processing. Cortisol levels were quantified using a commercially available enzyme immunoassay kit (Cayman Chemical Company, ref. 500360) following the manufacturer's instructions. Plasma samples were used directly into the kit without extraction, since it has been previously shown that there are no interferences of other putative immunoreactive substances with this kit in non-extracted plasma (Félix et al., 2013).

### Brain microdissection

After euthanasia, fish were quickly decapitated by cervical transection, the head removed, embedded in mounting media (OCT, Tissue teck) and rapidly frozen on dry ice. Brains were subsequently sectioned in coronal plane at 150 μm on a cryostat (Leica, CM 3050 S), and sections collected onto regular glass slides previously cleaned with 70% ethanol. The following brain nuclei of interest were selected for microdissection based on proposed homologies between the fish and the mammalian brain (O'Connell and Hofmann, 2011; Goodson and Kingsbury, 2013), which are indicated between brackets below, and identified in the zebrafish brain according to the available brain atlas (Wullimann et al., 1996): Dm, medial zone of the dorsal telencephalic area (basolateral amygdala); Dl, lateral zone of the dorsal telencephalic area (hippocampus); Vv, ventral nucleus of the ventral telencephalic area (septum); Vs, supracommissural nucleus of the ventral telencephalic area (subpallial amygdala); and POA, preoptic area (preoptic area plus paraventricular nucleus of the hypothalamus). Microdissection was performed with a modified 27G needle attached to a syringe under a stereoscope (Zeiss; Stemi 2000). Tissue was collected directly into lysis buffer (RNeasy Lipid Tissue Mini Kit-Qiagen) and stored at −80°C until mRNA extraction.

### Gene expression

Total RNA extraction was carried out immediately after thawing using the RNeasy Lipid Tissue Mini Kit (Qiagen) with some adjustments to the manufacturer's instructions (see the electronic Supplementary Material for details). RNA quality and concentration were estimated using NanoDrop 1000 spectrophotometer and cDNA was prepared using the iScript cDNA synthesis kit (Bio-Rad) according to manufacturer's instructions. Quantitative real-time PCR (qPCR) primers for the target genes (*bdnf*, *npas4, nlgn1, nlgn2, wnt3, neurod)* were designed at specific gene regions, therefore when necessary, homologous regions underlying gene family functions were excluded from primer design. However, for *nlgn2*, which is duplicated, both gene forms (i.e., *nlgn2a* and *nlgn2b*) where targeted by designing primers in homologous regions between the two sequences. The eukaryotic translation elongation factor 1 alpha 1, like 1 (*eef1a1l1*) was used as a reference gene. For each sample, transcript levels of candidate and reference gene were measured in 25 μl reactions on an Mx3000P qPCR system (Stratagene) using PerfeCTa SYBR Green FastMix, Low ROX (Quanta BioSciences). No-template controls for each primer mix were also included in each run (see the electronic Supplementary Material for further details). For the analysis, raw fluorescence data was submitted to PCR Miner (Zhao and Fernald, 2005) to calculate reaction efficiencies and cycle thresholds (CT) for each sample, and parameters subsequently used to determine the relative initial template concentration from 1/1(1+E)^∧^CT. Relative amount of mRNA in each sample was then normalized to the reference gene.

### Behavioral analysis

Behavioral analysis was performed using a computerized multi-event recorder (Observer XT, Noldus, Wageningen, Netherlands). The behaviors were divided into aggressive (bite, chase and strike) and submissive (freeze and flee), following the ethogram for zebrafish agonistic behavior (Oliveira et al., 2011). The following behavior variables were quantified: (1) latency for the first attack (i.e., time between the beginning of the recording period and the first bite); (2) fight resolution time (i.e., time needed for a social hierarchy to be established); (3) frequency of aggressive displays and (4) submissive behaviors, expressed in the last 5 min of the interaction, when winners and losers were easily distinguished allowing the recording of individual behavior.

### Statistical analysis

*T*-tests were used to compare the behavioral variables (i.e., latency for the first attack, fight resolution time, and overt aggression) between real opponent and mirror elicited fights. The effects of social treatment (Mirror-fight, Winner, Loser, Non-interacting) and brain nuclei (Dm, Dl, Vv, Vs, POA) in the expression of different genes (*bdnf*, *npas4, nlgn1, nlgn2, neurod, wnt3*) were evaluated using linear mixed models (LMM) with two random effects, one for the subjects and another for the dyads involved in real opponent interactions. The inclusion of the random effect for the dyadic real opponent interactions aims to address the fact that the data for Winners and Losers cannot be considered independent from each other since the behavior of each of them influences the other, and hence a matched-dyad analysis is more appropriate (Briffa and Elwood, 2010). To assess the assumptions of the mixed-effects models plots of the residuals, fitted values, and estimated random effects were used. Planned multiple comparisons analyses were then used to evaluate the effect of social treatment (Mirror-fight vs. Winners vs. Losers vs. Control) on gene expression at each brain nuclei. Effect sizes (Cohen's *d*_s_ for independent samples, and Cohen's *d*_z_ for dependent samples) for these comparisons were reported and reference effect size values (small: *d*>0.2, medium: *d*>0.5, and large: *d*>0.8) used to interpret the mean difference of the effect (Cohen, 1988). A one-way ANOVA with Welch correction for violation of homoscedasticity, followed by *post-hoc* tests (Tukey HSD) were used to compare cortisol levels among social treatments. Pearson correlations followed by Benjamini and Hochberg's method for *p*-value adjustment were also computed to test for associations between: (1) expressed behavior and gene expression; (2) expressed behavior and cortisol levels; and (3) cortisol levels and gene expression. To characterize the neurogenomic states elicited by each social treatment we used matrices of Pearson correlations, computed between the expression of each pair of candidate genes in each brain region. These correlations were considered as indicative of co-expression of candidate genes within each nucleus of the SDMN. Visual analyses of gene co-expression across treatments for each brain nucleus were performed using heatmaps of the correlation matrices. The occurrence of different patterns of gene co-expression associated with different social behavior states was assessed by testing the association between any two matrices within each nucleus, using the quadratic assignment procedure (QAP) correlation test with 5000 permutations (Borgatti et al., 2013). The null hypothesis of the QAP test is that there is no association between matrices, hence a non-significant *p*-value indicates that the correlation matrices are different.

Sample sizes varied either due to technical problems (i.e., problems with blood collection or video recordings) or to outlier values, identified for each condition with the generalized extreme studentized deviate procedure with a *p* = 0.05 and a maximum number of outliers of 20% of sample size. Statistical analyses were performed on R [(R Core Team, 2015); nlme (linear mixed models), multcomp (multiple comparisons), and Hmisc (correlations)], on SPSS v. 21 (one-way ANOVAs with Welch correction) and on UCINET 6 (Borgatti et al., 2002). For all tests the significance level used was *p* < 0.05.

### Ethics statement

The animal experimentation procedures used in this study followed the institutional guidelines for the use of animals in experimentation and were approved by the internal Ethics Committee of the Gulbenkian Institute of Science and by the National Veterinary Authority (Direção Geral de Alimentação e Veterinária, Portugal; permit number 8954).

## Results

### Behavior

**Figure 1:**
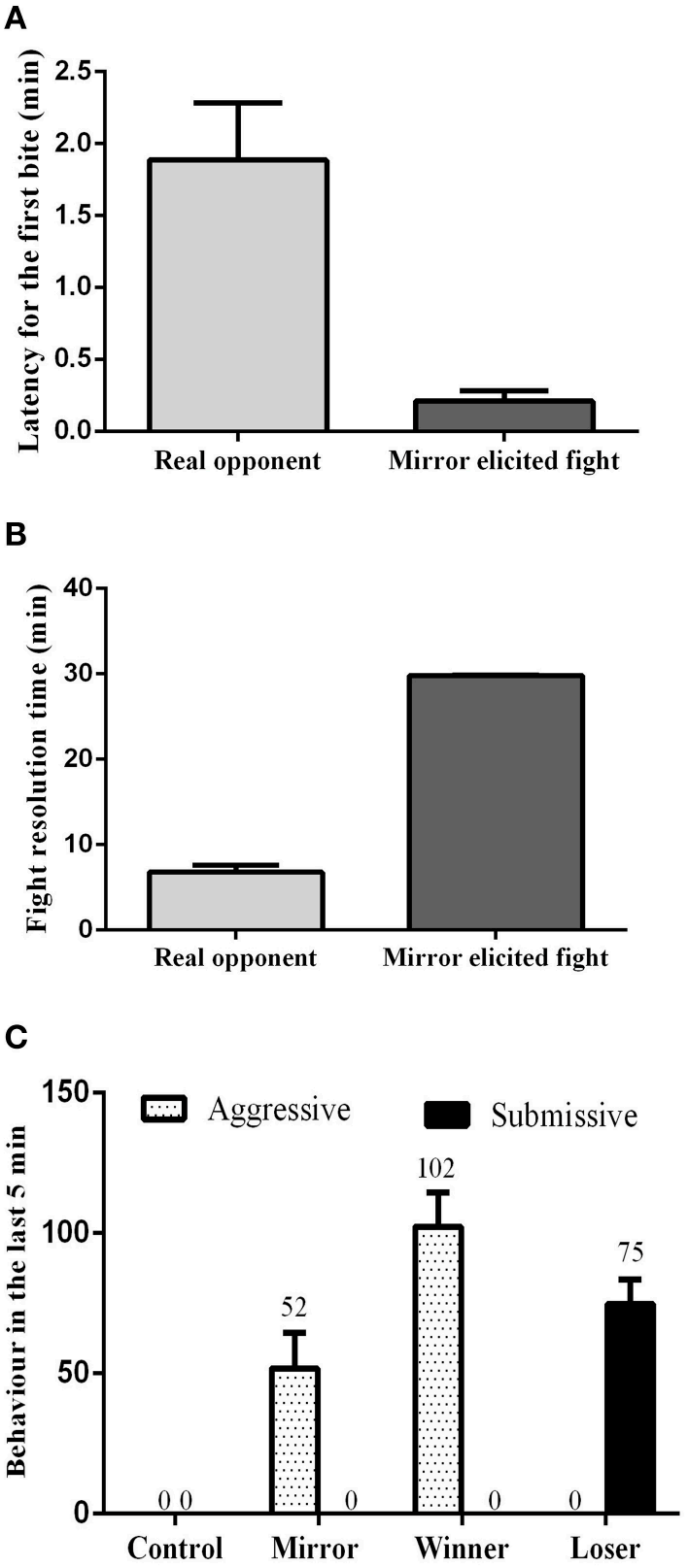
**Behavioral characterization of the different social phenotypes. (A)** Latency for the first bite; **(B)** Fight resolution time, the time required for the fight to be solved; **(C)** the frequency of aggressive and submissive behaviors expressed at the end of the agonistic interactions; error bars represent the standard error of the mean.

The influence of social treatment in aggressive behavior was measured in the pre-resolution phase by the latency to the first attack, and by the fight resolution time. During the assessment phase, the latency for the first bite was significantly lower in mirror-fighters (*t* = 4.15, *df* = 20, *p* < 0.001; Figure 1A), whereas the fight resolution time was significantly higher (*t* = 28.73, *df* = 20, *p* < 0.000; Figure 1B) when compared to real opponent interactions. Real opponent fights were solved in approximately 7 min, after which a dominance relationship was established. In contrast, mirror-fighters fought for the entire interaction period. In the post-resolution phase of real-opponent fights, aggressive behavior was only performed by winners, whereas losers only expressed submissive behavior (Figure 1C). In mirror-fighters aggressive behavior was exhibited during the entire interaction, and submissive behavior was never observed.

### Expression of *bdnf* across the SDMN

**Figure 2:**
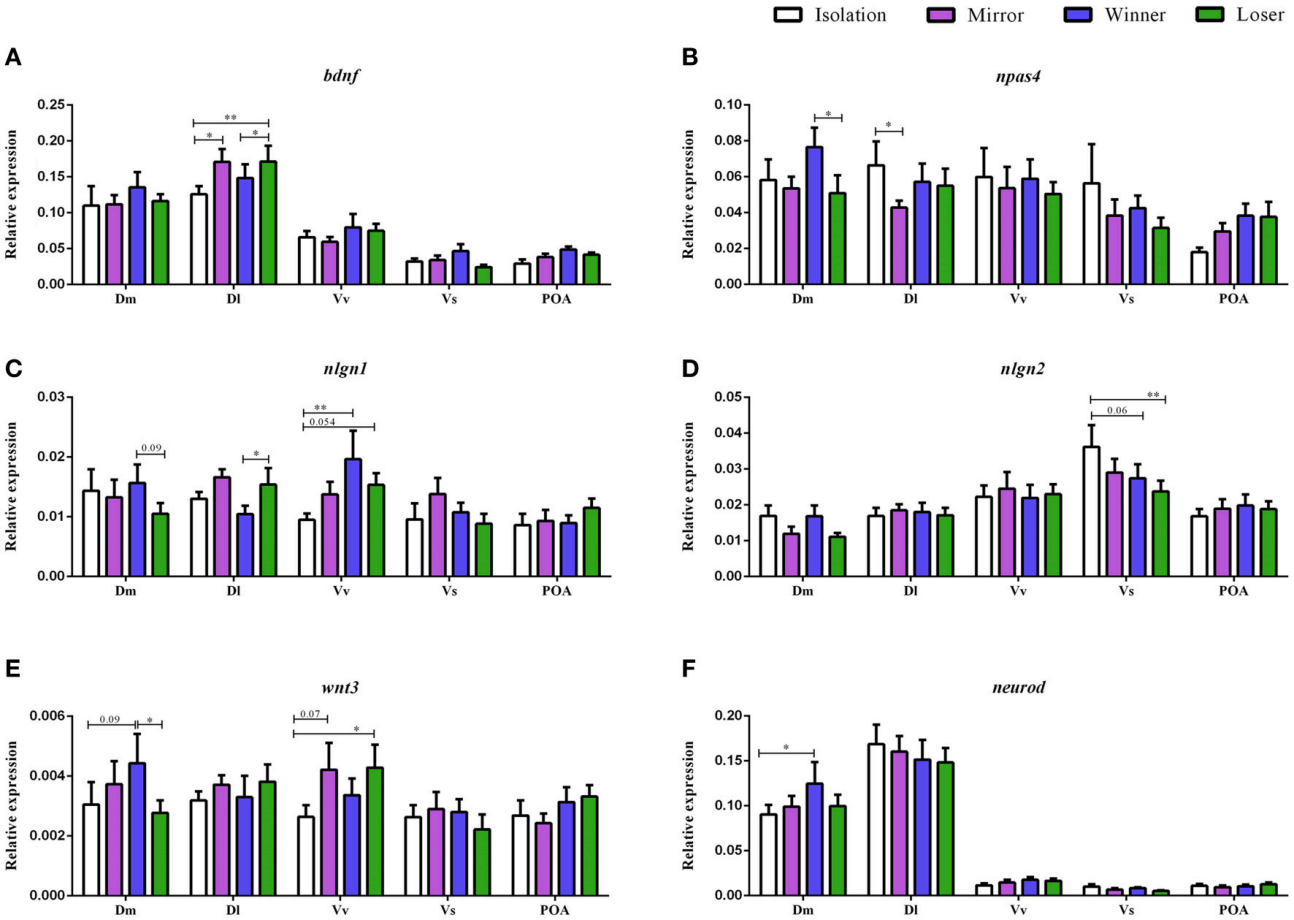
**Gene expression for the analyzed genes (*bdnf*, *npas4*, *nlgn1*, *nlgn2*, *wnt3*, and *neurod*) in different brain nuclei (Dm, medial zone of the dorsal telencephalic area; Dl, lateral zone of the dorsal telencephalic area; Vv, ventral nucleus of the ventral telencephalic area; Vs, supracommissural nucleus of the ventral telencephalic area; POA, preoptic area) for the different social phenotypes**. Control group is represented by the white bars, Mirror-fighters by the purple bars, Winners by the blue bars, and Loser by the green bars: **(A)** *bdnf* expression; **(B)** *npas4* expression; **(C)** *nlgn1* expression; **(D)** *nlgn2* expression; **(E)** *wnt3* expression; **(F)** *neurod* expression (normalized to *eef1a1l1* in); error bars represent the standard error of the mean. Asterisks indicate significant differences: ^*^p < 0.05; ^**^p < 0.01; using a planned comparisons test.

There was a significant main effect of brain nuclei on the expression of *bdnf* [*F*_(4, 152)_ = 80.75, *p* < 0.0001; Dl > Dm > Vv > POA > Vs, Table 1]. In contrast, no effects were found either for social treatment [*F*_(3, 7)_ = 0.83, *p* = 0.52], or for the interaction between social treatment and brain nuclei [*F*_(12, 152)_ = 0.89, *p* = 0.55]. Planned comparison analysis to evaluate the effect of social treatment on *bdnf* expression at each brain nuclei revealed that mirror-fighters and losers increased mRNA levels in the Dl when compared to the control group (*z* = 2.41, *p* = 0.015, *d*_*s*_ = 0.90; *z* = 2.80, *p* = 0.005, *d*_*s*_ = 0.77, respectively), and that Losers also increased in comparison to Winners (*z* = 1.99, *p* = 0.04, *d*_*z*_ = 0.48; Figure 2A).

**Table 1:** **Multiple comparisons analysis calculated using linear mixed models on the brain nuclei**.

	***bdnf***		***npas4***		***nlgn1***		***nlgn2***		***wnt3***		***neurod***	
**Brain nuclei**	***z*-value**	***p*-value**	***z*-value**	***p*-value**	***z*-value**	***p*-value**	***z*-value**	***p*-value**	***z*-value**	***p*-value**	***z*-value**	***p*-value**
Dm-Dl	−3.86	< 0.001	1.31	0.19	−0.67	0.50	−1.14	0.25	0.42	0.67	−6.75	< 0.0001
Dm-Vv	6.01	< 0.0001	1.61	0.10	−0.60	0.54	−3.82	< 0.001	0.24	0.80	10.73	< 0.0001
Dm-Vs	9.67	< 0.0001	3.86	< 0.001	1.97	< 0.05	−7.63	< 0.0001	2.36	< 0.05	11.37	< 0.0001
Dm-POA	9.14	< 0.0001	5.98	< 0.0001	3.09	< 0.01	−1.68	0.09	2.08	< 0.05	11.45	< 0.0001
Dl-Vv	9.79	< 0.0001	0.30	0.76	0.08	0.93	−2.74	< 0.01	−0.18	0.85	17.69	< 0.0001
Dl-Vs	13.31	< 0.0001	2.60	< 0.01	2.50	< 0.05	−6.67	< 0.0001	1.89	0.06	18.13	< 0.0001
Dl-POA	12.77	< 0.0001	4.71	< 0.0001	3.62	< 0.001	−0.55	0.57	1.60	0.10	18.47	< 0.0001
Vv-Vs	3.80	< 0.0001	2.35	< 0.05	2.48	< 0.05	−4.01	< 0.0001	2.09	< 0.05	0.89	0.37
Vv-POA	3.30	< 0.0001	4.44	< 0.0001	3.61	< 0.001	2.15	< 0.05	1.81	0.07	0.55	0.58
Vs-POA	0.47	< 0.0001	1.99	< 0.05	0.92	0.36	6.11	< 0.0001	0.35	0.72	−0.3	0.71

### Expression of *npas4* across the SDMN

There was a significant main effect of brain nuclei [*F*_(4, 145)_ = 10.13, *p* < 0.0001], with Dm, Dl, and Vv presenting higher mRNA levels than Vs and POA, and Vs was also significantly different from POA (Table 1). There were no significant effects of either social status or the interaction between the two factors [*F*_(3, 7)_ = 0.51, *p* = 0.69; *F*_(12, 145)_ = 1.02, *p* = 0.43] on the expression of *npas4*. Planned comparisons revealed an increase in *npas4* expression in the Dm of the Winners compared to Losers (*z* = −1.99, *p* = 0.046, *d*_*z*_ = 0.51) and a decrease in the mRNA levels of the Mirror-fighters in the Dl when compared to the control group (*z* = −2.21, *p* = 0.026, *d*_*s*_ = 0.80, Figure 2B).

### Expression of neuroligin genes across the SDMN

For *nlgn1* there was a main effect of brain nuclei [*F*_(4, 141)_ = 6.49, *p* < 0.001], with higher expression levels in Dm, Dl, and Vv compared to Vs and POA (Table 1). There was also an effect of the interaction between social treatment × brain nuclei [*F*_(12, 141)_ = 1.84, *p* = 0.04]. No effects were detected for social treatment [*F*_(3, 7)_ = 0.37, *p* = 0.77]. Planned comparison analysis revealed an increased in the Dl of Losers when compared to Winners (*z* = 2.06, *p* = 0.04, *d*_*z*_ = 0.57) and in the Vv of both Winners and Losers relative to controls (*z* = 2.74, *p* = 0.006, *d*_*s*_ = 0.84, *z* = 1.92, *p* = 0.054, *d*_*z*_ = 0.27). A close to significance response was also found in the Dm, where Losers decreased *nlgn1* expression in comparison to Winners (*z* = −1.66, *p* = 0.09, *d*_*s*_ = 0.56, Figure 2C).

For *nlgn2*, a main effect of brain nuclei was also detected [*F*_(4, 139)_ = 16.42, *p* < 0.0001], with major expression levels in the Vv and Vs compared to the other nuclei (Table 1), and no effects were found either for social treatment [*F*_(3, 7)_ = 0.06, *p* = 0.97] or for the interaction between social status and brain nuclei [*F*_(12, 139)_ = 1.10, *p* = 0.35]. Planned comparisons identified a response in Vs with a significant decrease in the mRNA levels of Losers, and a close to significance decrease of Winners when compared with the control group (*z* = −2.59, *p* = 0.009, *d*_*s*_ = 0.98; *z* = −1.84, *p* = 0.06, *d*_*s*_ = 0.65, Figure 2D).

### Expression of neurogenesis genes across the SDMN

There was a main effect of brain nuclei on the expression of both *wnt3* and *neurod* [*F*_(4, 144)_ = 3.11, *p* = 0.017; *F*_(4, 143)_ = 149.2, *p* < 0.001; respectively]. For *wnt3* higher abundance of transcripts was detected on Dm compared to Vs and POA, and also on Vv compared to Vs, for *neurod*, Dm, and Dl were the areas with higher expression levels (Table 1). There was no main effect for social treatment nor for the interaction between social treatment and brain nuclei for either of these two genes [*wnt3*, social treatment: *F*_(3, 7)_ = 0.58, *p* = 0.64; social status × brain nuclei: *F*_(12, 144)_ = 0.94, *p* = 0.51; *neurod*, social treatment: *F*_(3, 7)_ = 0.27, *p* = 0.84; social status × brain nuclei: *F*_(12, 143)_ = 0.57, *p* = 0.86]. Planned comparison analysis identified a significant increase in *wnt3* expression in the Dm of Winners in contrast with Losers (*z* = −2.07, *p* = 0.04, *d*_*s*_ = 0.44), and also a close to significance increase in the expression levels of Winners when compared to the control group (*z* = 1.68, *p* = 0.09, *d*_*s*_ = 0.38). There was also a marginally non-significant increase in the expression of *wnt3* in Vv both in Mirror fighters (*z* = 1.78, *p* = 0.07, *d*_*s*_ = 0.43) and in Losers (*z* = 2.16, *p* = 0.03, *d*_*s*_ = 0.43, Figure 2E) when compared to the control group. An increase in *neurod* expression was observed in the Dm of Winners when compared to the control group (*z* = 2.10, *p* = 0.03, *d*_*s*_ = 0.56, Figure 2F).

### Circulating cortisol levels

**Figure 3:**
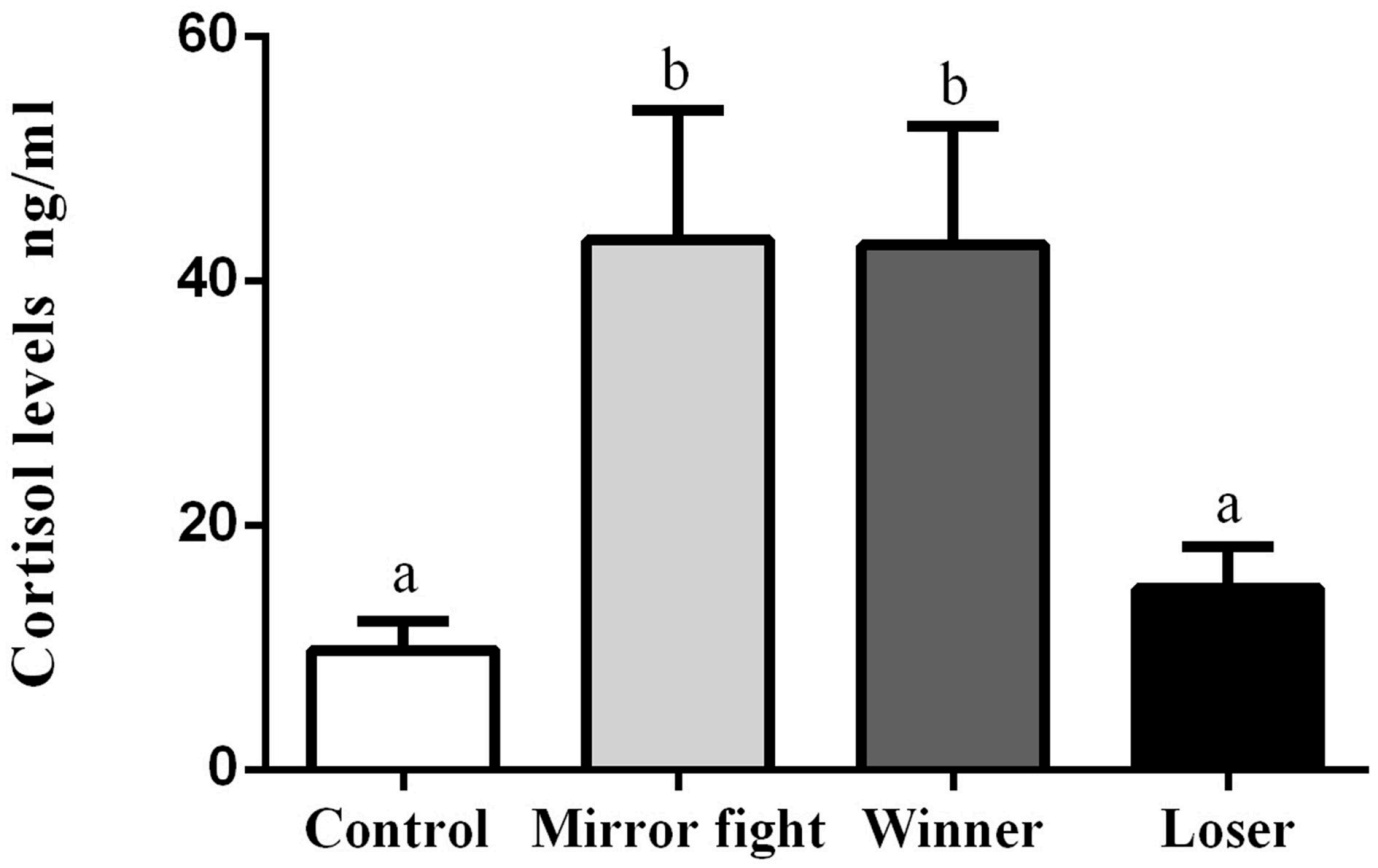
**Plasma cortisol concentrations (ng/ml) in the different social phenotypes 2 h post-interaction**. Error bars represent the standard error of the mean, and different letters indicate significant differences between the groups (*p* < 0.05).

Circulating levels of plasma cortisol sampled 2 h after the social interaction showed overall differences across groups [*F*_(3, 10.68)_ = 5.83, *p* = 0.013]. A *post-hoc* analysis revealed that Mirror-fighters and Winners had significantly higher cortisol levels than either Losers or individuals from the control group (Tukey HSD, *p* < 0.05, Figure 3).

### Association patterns between behavior, gene expression, and cortisol levels

After *p*-value adjustment there were no significant correlations between behavior and gene expression, behavior and cortisol levels, or cortisol levels and gene expression.

### Neurogenomic states across the SDMN

**Figure 4:**
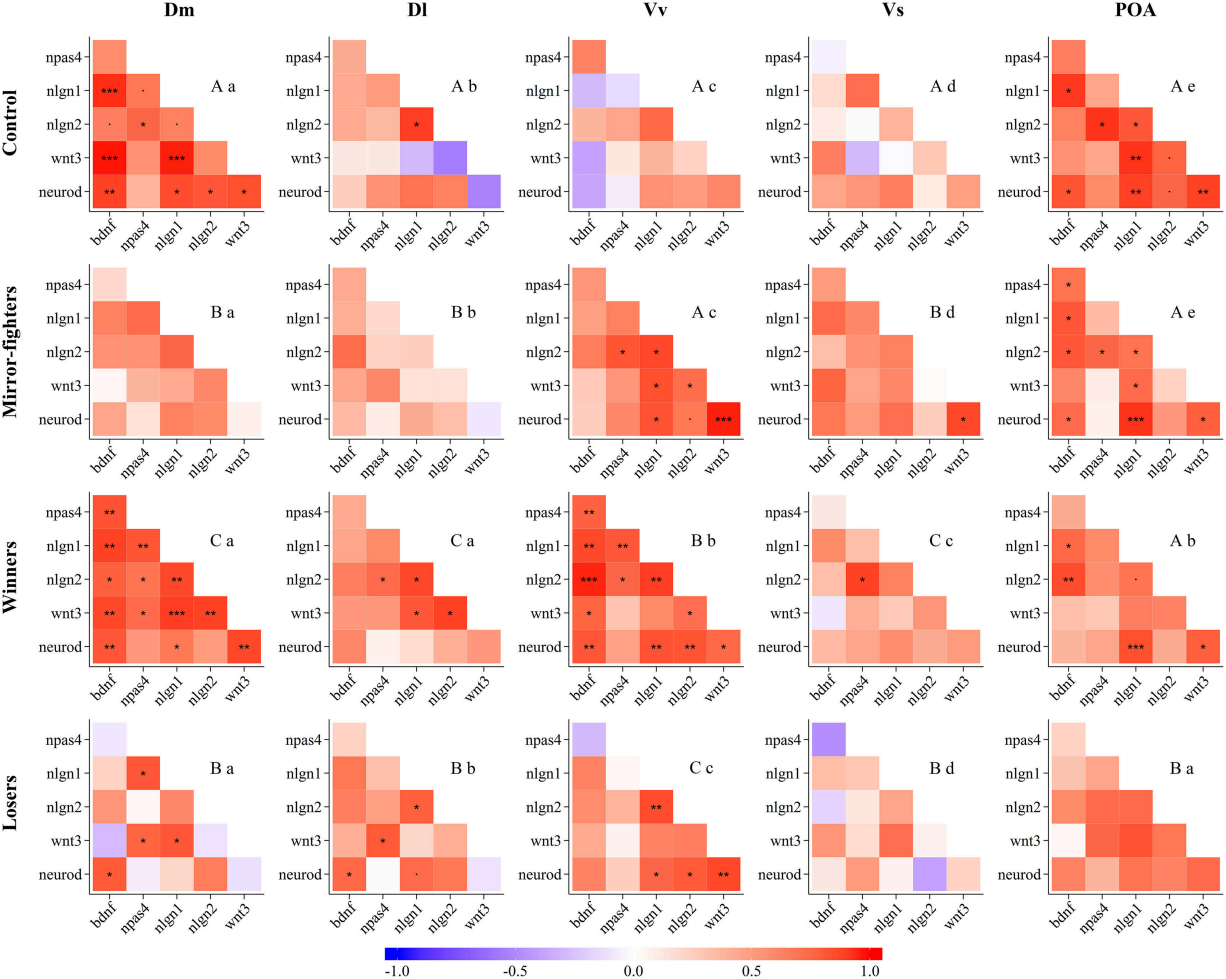
Neurogenomic states, as described by correlation (*r*) matrices of candidate genes expression in the different brain nuclei (Dm, medial zone of the dorsal telencephalic area; Dl, lateral zone of the dorsal telencephalic area; Vv, ventral nucleus of the ventral telencephalic area; Vs, supracommissural nucleus of the ventral telencephalic area; POA, preoptic area) for each social phenotype (Control, Mirror-fighters, Winners, Losers); Color scheme represents *r*-values from -1 (blue) to 1 (red); Asterisks indicate significant correlations after *p*-value adjustment ^*^p < 0.05; ^**^p < 0.01; ^***^p < 0.001; and the dot (.) close to significant results *p* < 0.1. Different capital letters indicate significantly different co-expression patterns among social treatments, and different small letters indicate significantly different co-expression patterns among brain nuclei, using the QAP correlation test.

Neurogenomic states, as captured by the co-expression patterns of the candidate genes, across brain regions, and across social treatments are presented in Figure 4 (see the electronic Supplementary Material for detailed information on QAP correlations and *p*-values, Tables S2 and S3, respectively). The neurogenomic states of the Dm, Dl, and Vs were similar between Mirror-fighters and Losers, and specific for Winners and for control fish. The neurogenomic state of Vv was similar between control fish and Mirror-fighters and specific for Winners and for Losers. Finally, the neurogenomic state of the POA was similar across all social treatments except for Losers. Regarding the comparison of neurogenomic states across brain regions within each social treatment, both Mirror-fighters, and control fish present different gene co-expression patterns across all brain regions. The Dm and the Dl, and the Vv and the POA present similar neurogenomic states in Winners, as well as the Dm and the POA in Losers.

## Discussion

The results of this study can be interpreted at two different levels: (1) the comparisons between each social treatment (i.e., Winners, Losers, and Mirror-fighters) and the reference group (i.e., controls: non-interacting individuals) allow the characterization of the neuromolecular response specific to each social treatment; (2) the comparisons of the different social treatments among themselves allow the interpretation of the source of the observed neuromolecular responses, according to the predictions presented at the end of the Introduction. In the Discussion of the results below we will address each of these two levels of analysis sequentially (see Table 2 for a summary of the univariate gene analyses).

**Table 2:** **Differential expressed genes in the SDM network in comparison with the control group and between Winners and Losers**.

	**Brain nuclei**				
**Social phenotype**	**Dm**	**Dl**	**Vv**	**Vs**	**POA**
Mirror–Control		*bdnf* 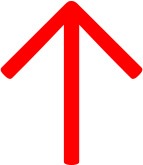	*wnt3* 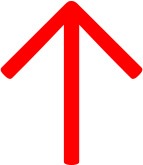 ^+^		
		*npas4* 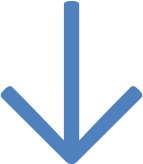			
Winner–Control	*neurod* 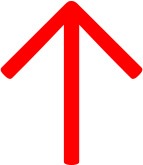		*nlgn1* 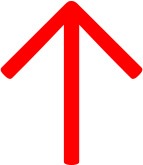	*nlgn2* 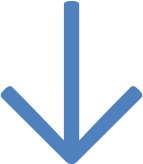 _+_	
	*wnt3* 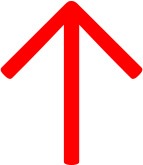 ^+^				
Loser–Control		*bdnf* 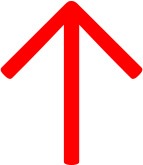	*wnt3* 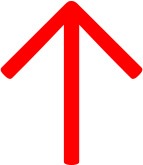 ^+^	*nlgn2* 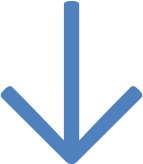	
					
Winner–Loser	*npas4* 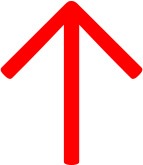	*bdnf* 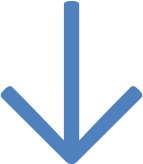			
	*nlgn1* 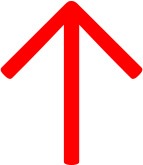 ^+^	*nlgn1* 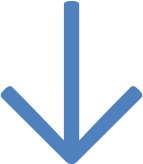			
	*wnt3* 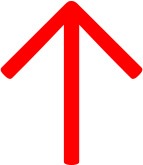				

*Red arrows (
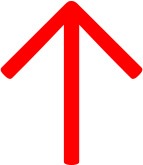
) indicates a significant increase in the expression, blue arrows (
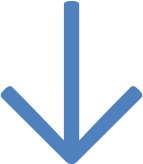
) a significant decrease in the expression with p < 0.05, and the plus (+) indicates close to significant results, p < 0.1. Dm, medial zone of the dorsal telencephalic area; Dl, lateral zone of the dorsal telencephalic area;Vv, ventral nucleus of the ventral telencephalic area; Vs, supracommissural nucleus of the ventral telencephalic area; POA, preoptic area*.

### Socially triggered neuroplasticity profiles for each social phenotype

Each social treatment was characterized by a specific neuromolecular pattern across the SDMN (Table 2; Figure 4). The Winner phenotype was characterized by an increase of the expression of neurogenesis genes (*wnt3* and *neurod*) in Dm (putative basolateral amygdala homolog), and of neuroligin genes (*nlgn1* and *nlgn2*) in Vv and Vs (putative homologs of the septum and subpallial amygdala, respectively; O'Connell and Hofmann, 2011; Goodson and Kingsbury, 2013). *Wnt* signaling is one of the main regulators of adult neurogenesis (Lie et al., 2005), and its expression has been shown to be activity-dependent and to be associated with LTP and synaptic plasticity (Chen et al., 2006). Moreover, *wnt3* mediates *neurod* activation via the canonical Wnt/β-catenin pathway (Kuwabara et al., 2009), which in turn is important for adult neurogenesis and survival of progenitor cells. Thus, the up-regulation of these two genes suggests a remodeling of Dm (basolateral amygdala) circuits in Winners. In mammals, the amygdala together with the dorsal hippocampus (putatively Dl in teleosts) are critically involved in the formation of contextual fear memory, with the former tracking emotional valence, and the latter forming a representation of the context (Zelikowsky et al., 2014). Thus our results, that suggest remodeling of Dm in the absence of regulation of the Dl, may indicate changes at the level of valence encoding without context modulation in Winners. The differential expression of neuroligin genes in Winners is also interesting. These are post-synaptic cell adhesion molecules involved in synaptogenesis and synapse maturation in an activity-dependent fashion, by binding to pre-synaptic neuroxins (Scheiffele et al., 2000). Neuroligins also affect synaptic function by recruiting and stabilizing key synaptic components, such as neurotransmitter receptors and channels. Both mammals and fish express 4 neuroligin genes (in zebrafish *nlgn* 2–4 are duplicated) with *nlgn1* and *nlgn2* exclusively expressed in excitatory and inhibitory synapses respectively, whereas *nlgn3* and *nlgn4* may be present in both (Südhof, 2008; Rissone et al., 2010). The region-specific effects of social phenotype on *nlgn1* in Vv and of *nlgn2* in Vs overall match previously described distribution of these genes in the CNS of zebrafish (Davey et al., 2010). In Winners the increase of *nlgn1* mRNA levels in the septum homolog (Vv) may be associated with the storage of associative memories related to status-acquisition, given the role *nlgn1* in excitatory glutamatergic synapses in associative learning (Kim et al., 2008). The decrease in *nlgn2* expression in the subpallial amygdala (Vs) maybe associated with behavioral desinhibition in Winners related to a down-regulation of GABAergic synapses. Indeed, the manipulation of *nlgn2* has been shown to increase anxiety-like behaviors in mice (Hines et al., 2008). In terms of neurogenomic state, Winners showed a unique pattern of gene co-expression across all the SDMN nodes sampled in this study (Figure 4).

Losers were characterized by an increase of the expression of *bdnf* in Dl and of the neurogenesis gene *wnt3* in Vv, and by a decrease in the expression of the synaptic gene *nlgn2* in Vs. Interestingly, Mirror-fighters showed a neuromolecular pattern that partially overlapped with that of Losers: an increased expression of *bdnf* in Dl and of *wnt3* in Vv. Additionally Mirror-fighters also exhibited a decrease of *npas4* in Dl. BDNF is a key molecule involved in the control of neuronal differentiation and survival, synapse formation, and in the regulation of activity-dependent changes in synapse structure and function (Park and Poo, 2013). In particular, BDNF signaling in the mammalian hippocampus has been implicated in learning and memory through its effect on long-term potentiation and depression (Kovalchuk et al., 2002; Egan et al., 2003; Park and Poo, 2013). Thus, the region-specific effect of social treatment on *bdnf* expression localized in the fish homolog of the tetrapod hippocampus (Dl, O'Connell and Hofmann, 2011) is not surprising, and may suggest an involvement of Dl in social memory in both Losers and Mirror-fighters, that would thus recognize dominant individuals. In this respect it is worth noting that these two social phenotypes are the ones that have an opponent that expresses high levels of aggressive behavior, and this might be a key feature to trigger social recognition mechanisms in an aggressive context. This hypothesis is also supported by the known role of hippocampus-dependent memory in social recognition in mice (Kogan et al., 2000). *Wnt3* was also up-regulated in the Vv of both Losers and Mirror-fighters, pointing to the occurrence of structural reorganization processes in this area in both social phenotypes. In mammals the lateral septum is involved in fear suppression (Thomas et al., 2013) as well as in the extinction of social fear conditioning (Zoicas et al., 2014), and a variety of anxiolytic or antidepressant drugs activate it (Thomas et al., 2005; Rodrìguez-Landa et al., 2007). Thus, for both phenotypes, *wnt3* mediated changes in this septal region may be related with anxiety and fear control: in Losers due to the defeat, and in Mirror-fighters due to the anxiety of an unsolved fight. The distinction between these two social phenotypes comes from a down-regulation of *npas4* in the Dl of Mirror-fighters, and of *nlgn2* in the Vs of Losers. *Npas4* is an activity-dependent transcription factor expressed in inhibitory and excitatory synapses that modulates the excitatory-inhibitory balance within neural circuits that are being activated (Lin et al., 2008). It has a cell-type-specific transcription gene program that induces inhibitory outputs on both cell types decreasing circuit activity; in excitatory neurons by the expression of synaptic connectivity regulators (e.g., *bdnf*), and in inhibitory neurons by a different gene set (Spiegel et al., 2014). *Npas4* has recently been implicated in the formation of contextual memories in the hippocampus (Ramamoorthi et al., 2011). Thus, the decreased expression of *npas4* in Mirror-fighters may indicate a decline in synaptic inhibition, as well as a lack of contextual memory formation. On the other hand, the decrease in the expression of *nlgn2* in the Vs of Losers, similar to what happens in Winners, may be related with anxiety behaviors as well. Despite the fact that Winners and Losers express different behavioral phenotypes, anxiety-like behaviors are expected to occur in both phenotypes after an agonistic interaction.

Finally, Losers and Mirror-fighters also showed a common pattern of gene co-expression in the Dm, Dl, and Vs, suggesting the activation of overall similar neuroplasticity mechanisms in these three brain regions, but of unique ones in the remaining two regions (i.e., Vv and POA). Thus, each social phenotype (i.e., Winners, Losers and Mirror-fighters) exhibited specific overall neurogenomic states across the SDMN, which supports previous results using a genome-wide transcriptional profiling of these three phenotypes (Oliveira et al., 2016).

### Status-specific and fighting triggered neuromolecular states of the SDMN

According to the rational described above (see Introduction) changes in gene expression in Winners/Losers shared by Mirror-fighters in relation to the reference group should reflect neuromolecular changes triggered by fighting behavior, whereas the same changes not shared by Mirror-fighters should reflect status-specific neurogenomic states. Accordingly, the increase in neurogenesis genes in Dm and the changes in synaptic genes in Vv and Vs observed in Winners should be seen as status-specific. Similarly, the decrease in the expression of *npas4* in Mirror-fighters' Dl should be seen as specific of this phenotype. In contrast, there were no status-specific neuromolecular changes in Losers. Neuromolecular changes shared by different social treatments that hence reflect fighting behavior, rather than status-specific neurogenomic states included: (1) the decrease of *nlgn2* in Vs both in Winners and Losers; and (2) the increase in *bdnf* in Dl and the increase of *wnt3* in Vv observed both in Losers and Mirror-fighters. Because the shared experiences between Winners and Losers and between Losers and Mirror-fighters are different, one may infer what aspect of the agonistic behavior is driving these changes. In the former case, both social phenotypes (i.e., Winners-Losers) share the expression of displaying behavior during the initial phase of the fights when individuals assess each other (Oliveira et al., 2011). Only after this assessment phase an asymmetry in agonistic behavior emerges and Winners chase and attack Losers that only express submissive behavior in this post-resolution phase of the fight (Oliveira et al., 2011). Therefore, the shared pattern of gene expression between Winners and Losers (i.e., decreased expression of *nlgn2* in Vs) most probably reflects the similar behavioral display patterns expressed by both phenotypes in the initial phases of the fights. In the latter case, the behavioral experience shared by both Losers and Mirror-fighters is not the behavior expressed, which is aggressive in Mirror-fighters and Submissive in Losers, but rather the behavior observed in the opponent, which is the aggressive behavior displayed either by the real opponent in the case of Losers or by the mirror-image in the case of Mirror-fighters. Therefore, the shared neuromolecular pattern observed in Losers and Mirror-fighters (i.e., increase in *bdnf* in Dl and the increase of *wnt3* in Vv) most probably is triggered by the perception of aggressive behavior in a fighting opponent.

As a result of the increases/decreases of expression of the different genes in relation to the reference group discussed above, significant differences between social treatments may also emerge. In this study such differences were only observed between Winners and Losers in Dm for *npas4, nlgn1* and *wnt3*, and in Dl for *bdnf* and for *nlgn1*. These differences between social phenotypes are difficult to interpret since they may result from variations in each of the two phenotypes that are being compared in relation to the reference group.

### Brain region specific neuroplasticity

This study also allowed to test if there are region specific neuroplasticity mechanisms across the SDMN in relation to the expression of social behavior. Such regional variation was indeed observed with some neuroplasticity mechanisms being associated with social behavior only at certain regions of the SDMN. In the basolateral amygdala homolog (Dm) only neurogenesis genes (*wnt3* and *neurod*) were associated with one of the social phenotypes (Winners). In the hippocampus homolog (Dl), only genes involved in molecular processes related to memory (*bdnf, npas4*) were associated with social phenotypes (Losers, Mirrror-fighters). In the septum homolog (Vv), genes related to cell proliferation (*wnt3*), and to synaptic plasticity (*nlgn1*) were associated with social phenotypes (Losers and Mirror fighters, and Winners, respectively). In the subpallial amygdala homolog (Vs) only genes involved in synaptic plasticity (*nlgn1, nlgn2*) were associated with social phenotypes (Winners and Losers). No neuroplasticity changes were observed in the POA. Finally, gene co-expression patterns were unique to each brain region within each social treatment (Figure 4), except for shared co-expression patterns between Dm and Dl in Winners and between Dm and POA in Losers, suggesting a coupling of neuroplasticity mechanisms between these areas in the mentioned social phenotypes.

In summary our study presents the first experimental evidence that after an acute agonistic interaction different neuroplasticity mechanisms are activated in a brain-region specific fashion, which parallel the social-status specific changes in social behavior observed. This indicates that social plasticity relies on multiple neuroplasticity mechanisms across the SDMN, and that there is not a single neuromolecular module underlying this type of behavioral flexibility.

## Author contributions

MT and RO designed the experiment; MT and SC performed the experiment; MT analyzed the data; MT and RO wrote the paper with contributions from all authors.

## Funding

This study was funded by the EU project “Copewell” (Grant no: 265957) and by a grant from Fundação para a Ciência e a Tecnologia (FCT, EXCL/BIA-ANM/0549/2012 awarded to RO). MT and SC were supported by individual fellowships from FCT (FCT, SFRH/BD/44848/2008 and SFRH/BD/89072/2012, respectively).

### Conflict of interest statement

The authors declare that the research was conducted in the absence of any commercial or financial relationships that could be construed as a potential conflict of interest.
