# Supplementary material for: Social Plasticity Relies on Different Neuroplasticity Mechanisms across the Brain Social Decision-Making Network in Zebrafish
Source: Front Behav Neurosci. 2016 Feb 16;10:16. doi: 10.3389/fnbeh.2016.00016 (PMC4754415; doi:10.3389/fnbeh.2016.00016)
Supplement: Supplementary file 1 [file Presentation1.PDF]

Frontiers in behavioral Neuroscience

Electronic Supplementary Material

Social plasticity relies on different neuroplasticity mechanisms across the social decision making network in zebrafish

Magda C. Teles, Sara D. Cardoso and Rui F. Oliveira

Correspondence to:

Rui F. Oliveira,
Integrative Behavioural Biology
Instituto Gulbenkian de Ciência,
Rua da Quinta Grande 6,
2780-156 Oeiras, Portugal
ruiol@ispa.pt

Rna extraction

Tissue was homogenized in quiazol lysis reagent by vortex followed by an incubation of 7 min at room temperature (RT). Chloroform was added in a proportion of 1:2 and the sample incubated at RT for 5 min. Samples were subsequently centrifuge at 13000 x *g* for 20 min at 4ºC, and the upper aqueous phase transferred to new tube where 1 volume of 70% ethanol was added. This mixture was transferred to an RNEasy column, remained 5 min at RT, and was centrifuged for 1 min at 9000 x *g*. A sequence of buffers was added to the Rneasy column according to the manufacturer's instructions, and RNA eluted with 25 µl of RNAse-free water

Primers design and quantitive real-time PCR (qPCR)

Primers were designed using Primer3web (Untergasser et al., 2012), with parameters adjusted to avoid dimer and hairpin formation, and their specificity confirmed by

Primer-BLAST search (NCBI). Primers were commercially synthetized by Eurofins MWG Operon. Thermocycling conditions were 5 min at 95°C followed by 40 cycles of: 95°C, primer specific annealing temperature (see Table S1for detailed information), and 72°C for 30 s each. A melting curve program from 55°C to 95°C with 0.5°C change in 10s intervals concluded the cycling protocol. The presence of single peaks in melting curves and gel electrophoresis performed on the PCR products confirmed the specificity of each primer pair. The identity of PCR products for each gene was also verified by DNA sequencing.

For each sample, transcript levels of candidate and reference gene were measured in 25 µl reactions and primers used at a concentration of 0.4 µM.

Table S1 – List of genes and corresponding primer sequences and parameters for quantitive real-time PCR (qPCR).

Gene name	Abbreviation	Accession No.	Primer Sequences (5’ → 3’)	Annealing temperature (°C)	Amplicon length (bp)
eukaryotic translation elongation factor 1 alpha 1, like 1	*eef1a1l1*	NM_131263	F-CAAGGAAGTCAGCGCATACA<br>R-TCTTCCATCCCTTGAACCAG	60	134
brain-derived neurotrophic factor	*bdnf*	NM_131595	F- GCTGCCGAGGAATAGACAAG<br>R- CTGCCCCTCTTAATGGTCAA	58	157
neuronal PAS domain protein 4a	*npas4a*	NM_001045321	F- GACACGGGTTGAGAATGGTT<br>R- GCACCAAGCACCCTGTAAAT	59	165
wingless-type MMTV integration site family, member 3	*wnt3*	NM_001114552	F- CTGTTGGGGGACTACCTGAA<br>R- GGCGTATTTGGCTCGTAGTG	57	108
neuronal differentiation 1	*neurod1*	NM_130978	F- AAGTCAGATCCCTGCGTCAT<br>R- GGGAATTGTGCAACTCTGC	63	185
neuroligin 1	*nlgn1*	NM_001142265	F- TCAACGAGGTCAGCCAGATA<br>R- TGAAGCACCGACAGCAATAG	59	221
neuroligin 2 a/b	*nlgn2a*<br>*nlgn2b*	NM_001166336<br>NM_001166329	F- GTCTGCCAAAGGGAACTATG<br>R- ATGGTGGGACAGGATGAGTA	59	157

F - primer forward; R - primer reverse

Table S2 – Quadratic assignment procedure (QAP) correlation test for the different brain nuclei. Dm, medial zone of the dorsal telencephalic area; Dl, lateral zone of the dorsal telencephalic area; Vv, ventral nucleus of the ventral telencephalic area; Vs, supracommissural nucleus of the ventral telencephalic area; POA, preoptic area. Significant correlations are indicated in bold for p < 0.05.

	**Brain nuclei**				
**Social phenotypes**	**Dm**	**Dl**	**Vv**	**Vs**	**POA**
I-M	r=0.012, p=0.525	r=0.309, p=0.126	**r=0.810, p=0.003**	r=0.401, p=0.075	**r=0.677, p=0.008**
I-W	r=0.361,p= 0.127	r=-0.403 p=0.107	r=0.152, p=0.270	r=-0.125, p=0.337	**r=0.515, p=0.026**
I-L	r=0.09, p=0.388	r=0.463, p=0.060	r=0.207, p=0.200	r=0.332, p=0.108	r=0.430, p=0.067
M-W	r=0.01, p=0.441	r=-0.08, p=0.389	r=0.066, p=0.396	r=-0.088, p=0.405	**r=0.549, p=0.029**
M-L	**r=0.571, p=0.01**	**r=0.681, p=0.017**	r=0.386, p=0.168	**r=0.472, p=0.042**	r=0.071, p=0.390
W-L	r=0.03, p=0.460	r=0.171, p=0.223	r=0.406, p=0.169	r=0.012, p=0.488	r=0.,360 p=0.097

Table S3- Quadratic assignment procedure (QAP) correlation test for the different social phenotypes. Dm, medial zone of the dorsal telencephalic area; Dl, lateral zone of the dorsal telencephalic area; Vv, ventral nucleus of the ventral telencephalic area; Vs, supracommissural nucleus of the ventral telencephalic area; POA, preoptic area. Significant correlations are indicated in bold for p < 0.05.

	**Social phenotypes**			
**Brain nuclei**	**Isolation**	**Mirror**	**Winner**	**Loser**
Dm-Dl	r=-0.182, p=0.320	r=0.155, p=0.324	**r=0.646, p=0.015**	r=0.507, p=0.068
Dm-Vv	r=-0.210, p=0.222	r=0.259, p=0.236	r=0.234, p=0.230	r=0.081, p=0.372
Dm-Vs	r=0.159, p=0.294	r=-0.304, p=0.187	r=-0.230, p=0.226	r=0.,039 p=0.430
Dm-POA	r=0.528, p=0.055	r=0.144, p=0.350	r=0.241, p=0.203	**r=0.475, p=0.020**
Dl-Vv	r=0.048, p=0.397	r=-0.491, p=0.054	r=0.194, p=0.298	r=0.213, p=0.254
Dl-Vs	r=-0.152, p=0.281	r=-0.177, p=0.264	r=0.130, p=0.348	r=-0.213, p=0.256
Dl-POA	r=-0.124, p=0.353	r=0.120, p=0.309	r=0.119, p=0.351	r=0.189, p=0.263
Vv-Vs	r=-0.323, p=0.112	r=-0.060, p=0.458	r=0.063, p=0.387	r=0.194, p=0.316
Vv-POA	r=0.345, p=0.123	r=0.196, p=0.458	**r=0.523, p=0.025**	r=0.454, p=0.132

Vs-POA	r=-0.052, p=0.415	r=0.488, p=0.254	r=0.445, p=0.052	r=0.034, p=0.462

References

Untergasser, A., Cutcutache, I., Koressaar, T., Ye, J., Faircloth, B. C., Remm, M., et al. (2012). Primer3–new capabilities and interfaces. *Nucleic Acids Res.* 40, e115. doi:10.1093/nar/gks596.
